# In vivo study of different methods for diagnosing pit and fissure caries

**DOI:** 10.4317/jced.52347

**Published:** 2015-07-01

**Authors:** María Melo, Agustín Pascual, Isabel Camps, Ángel Del Campo

**Affiliations:** 1 Associate Professor, Department of Dental Materials, University of Valencia; 2 Assistant Professor, Department of Dental Materials, University of Valencia; 3 Collaborating Professor, Department of Dental Materials, European University of Valencia. Valencia, Spain

## Abstract

**Background:**

In recent years the early detection of such caries has gained importance, since it may avoid unnecessary dental tissue damage and allow minimally invasive dental treatment. A study is made of 5 systems for diagnosing caries: traditional visual and tactile methods, DIAGNOdent, VistaProof and CarieScan.

**Material and Methods:**

A prospective study was made in the Department of Stomatology, Dental Pathology and Therapeutics Teaching unit of the University of Valencia (Valencia, Spain), involving the analysis of 32 teeth (molars or premolars of both arches scheduled for filling or for use as posts in dental bridges) in 28 patients. The following caries diagnostic methods were applied: visual, tactile, DIAGNOdent (KAvo, Biberach, Germany), VistaProof (Dürr Dental AG, Bietigheim-Bissingen, Germany) and CarieScan (IDMoS Dental Systems, Dundee, Scotland, United Kingdom). Fissurotomy was subsequently performed for histological validation.

**Results:**

Visual inspection showed an area under the receiver operating characteristic curve (AUC-ROC) of 0.75, with a sensitivity and specificity of 0.75. Tactile diagnosis in turn showed AUC = 0.714, with maximum sensitivity (100%) and a specificity of 42.9%. DIAGNOdent (cutoff point 22.5) and VistaProof (cutoff point 1.1) showed AUC = 0.969, while CarieScan (cutoff point 21.5) presented AUC = 0.973. These latter three methods all had a sensitivity of over 92%. The specificity of DIAGNOdent was maximum, while that of CarieScan and VistaProof was 75%.

**Conclusions:**

The emergent methods in the diagnosis of caries (DIAGNOdent, VistaProof and CarieScan) yielded similar results, and in all cases proved superior to the traditional visual and tactile methods. DIAGNOdent was seen to be the most effective technique, followed by CarieScan and VistaProof.

** Key words:**Caries diagnosis, emergent diagnostic methods, fluorescence, electrical impedance, minimally invasive dentistry.

## Introduction

Occlusal caries of the molars and premolars are the most frequent type of caries (1-2). In recent years the early detection of such caries has gained importance, since it may avoid unnecessary dental tissue damage and allow minimally invasive dental treatment (2-3).

This “*in vivo*” study compares the conventional visual and tactile methods for the diagnosis of caries (4-9) with three emergent diagnostic systems.

The visual-tactile technique is the most widely used option in clinical practice (5), though previous studies have found it to have variable sensitivity and specificity (5-8). The validity of using a probe for the diagnosis of occlusal caries has been questioned by a number of authors (1-2,6), due to the variability of fissure shape, probe shape or the pressure exerted by the examiner. All these factors can influence the results obtained.

The CarieScan system (IDMoS, Dundee, Scotland) is based on the use of alternating current impedance spectroscopy (ACIST) for the identification of incipient caries (9). In the presence of demineralization, dental permeability is seen to increase (10), in the same way that a radiotransparency is observed on the X-rays (11). Such permeability in turn is related to the electrical resistance of the tooth; as a result, the physical changes induced by the development of caries can be identified and quantified by measuring this electrical phenomenon (9,10). The different studies published to date have reported good sensitivity and specificity performance with this technique (9-10).

Fluorescence for the detection of carious lesions has been used for over two decades (5-8). The DIAGNOdent (KAvo, Biberach, Germany) and VistaProof systems (Dürr Dental, Bietigheim-Bissingen, Germany) are based on this phenomenon (12-15). When a tooth is irradiated with light, the latter is absorbed by the organic and inorganic substances present in the dental tissues, and by bacterial metabolites. Different “*in vivo*” and "in vitro" studies have yielded variable results with the first of the mentioned laser-induced fluorescence systems (5-8,12-13). The VistaProof technique in turn detects protoporphyrin IX (PP9) activity, with favorable sensitivity and specificity performance (14-15).

## Material and Methods

A prospective “*in vivo*” study was made in the Department of Stomatology, Dental Pathology and Therapeutics Teaching unit of the University of Valencia (Valencia, Spain), involving the analysis of 32 teeth (molars or premolars of both arches scheduled for filling or for use as posts in dental bridges) in 28 patients. Teeth with previous restorations or with fissure sealants were excluded, as were hypoplastic teeth or teeth with fluorosis or presenting amelogenesis.

The study was approved by the Clinical Research Ethics Committee of the University of Valencia (Valencia, Spain), and written informed consent was obtained from all the patients.

Regarding the traditional techniques for the diagnosis of caries, the visual method was carried out on the dry tooth, without magnification. The tactile diagnosis in turn was made by gently moving a TU 17/23 exploratory probe (Hu-Friedy, Chicago, IL, USA) over the dental surface. The emergent diagnostic methods were CarieScan (IDMoS, Dundee, Scotland), DIAGNOdent (KAvo, Biberach, Germany) and VistaProof (Dürr Dental, Bietigheim-Bissingen, Germany). These methods were applied after relative isolation and drying of the study teeth during four seconds using the system air syringe (16,18). In the case of the CarieScan system, the soft tissues clip was placed on the lip of the patient. The sensor was positioned on the occlusal surface of the tooth, and the “enter” button was pressed to start measurement. The numerical result obtained was then correlated to a code in the color pyramid. The DIAGNOdent and VistaProof systems are fluorescence-based techniques. In the case of the former, we fitted and calibrated the optical probe A, which was then placed in contact with the target tooth, without applying pressure. The reading obtained was scored between 0-99 (17-18). In turn, the VistaProof system uses an intraoral camera with a light-emitting diode (LED) unit that emits light at a wavelength of 405 nm, employed for detecting PP9 fluorescence. The image was captured by a sensor, and algorithms were used to generate visual representations with numerical data on the computed screen (15,19).

Fissurotomy was performed using round diamond drills measuring 0.5, 1 and 1.5 mm in diameter (Komet).

We first cleaned the teeth with the prophylaxis kit. Visual and tactile inspection was made under optimum conditions, according to the indications of the ICDAS II expert committee. On identifying caries lesions we also recorded their estimated depth (E1: superficial half of the enamel layer; E2: internal half of the enamel layer, reaching the amelodentinal junction; D1: external half of the dentinal layer; D2: carious lesions extending beyond the external half of the dentinal layer). This classification is a modification of that developed by Ekstrand *et al.* (4), already used by other authors (5).

Fissurotomy was taken as the gold standard for validation purposes. The final lesion depth was that considered in the study, and was corroborated visually and by means of the tip of the exploratory probe, assessing the hardness of the depth of the open fissure.

The data were entered on a spreadsheet, and were processed using the SPSS version 15.0 statistical package (SPSS Inc., Chicago, IL, USA).

Tables were generated for the descriptive analysis, with the calculation of sensitivity, specificity and positive and negative predictive values. The receiver operating characteristic (ROC) curves of each of the studied methods were also plotted. The bivariate analysis was performed using the Mann-Whitney U-test for several independent samples, to determine whether the distribution of values was homogeneous in one or two groups, e.g., teeth with or without caries, according to the ICDAS histological score or ICDAS numerical classification from 0-5. The Kappa concordance index in turn was used to assess agreement between the results of the visual diagnostic technique and the biopsy (all with a significance level of 5%).

## Results

According to the visual diagnostic technique, 68.7% of the analyzed teeth had caries. The positive and negative predictive values were 95.5% and 30%, respectively ( Table 1), and sensitivity and specificity was 75%, i.e., moderate. The significance obtained for the ROC curve (*p*=0.111) can be regarded as the same as for the main diagonal (random classification curve).

**Table 1:**
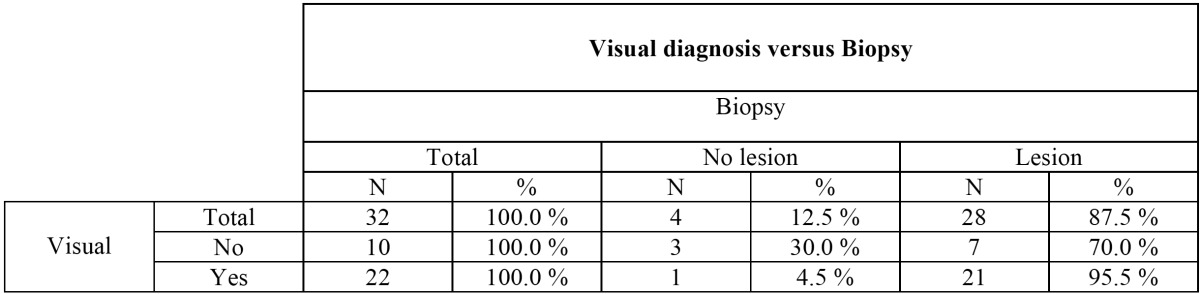
Relationship between the visual diagnosis of caries and the actual extent of the lesion as evidenced by fissurotomy.

In the case of the tactile diagnostic technique, 37.5% of the analyzed teeth were considered to have caries. The specificity and positive predictive values were maximum (100%), while the sensitivity and negative predictive values were 42.9% and 20%, respectively. The significance in this case was *p*=0.171.

The DIAGNOdent findings were closely correlated to the fissurotomy results, with a median of 15.0 among the teeth without caries according to the biopsy findings and a median of 46.5 among the teeth with caries ( Table 2). This difference was significant according to both the Mann-Whitney U-test and the Kruskal-Wallis test (*p* < 0.001).

**Table 2:**
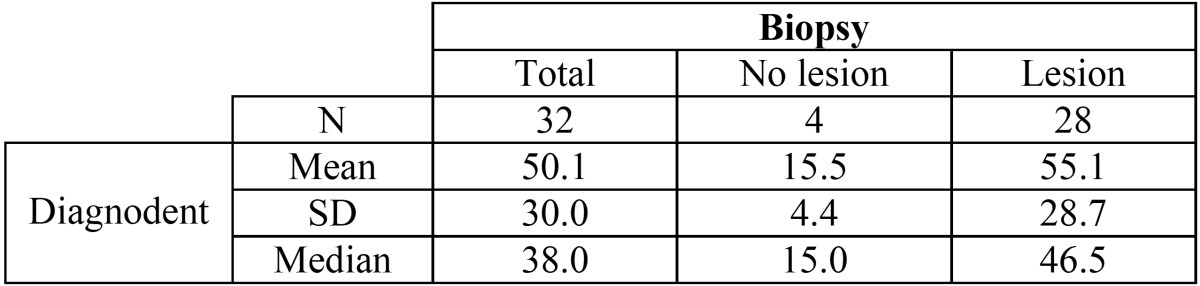
Relationship between the values obtained with the DIAGNOdent method and the actual extent of the lesion.

The area under the ROC curve was 0.969, showing significant differentiation from the main diagonal. The optimum cutoff point was 22.5. Accordingly, the sensitivity was 96.4%, specificity and positive predictive value 100%, and negative predictive value 80%.

**Figure 1:**
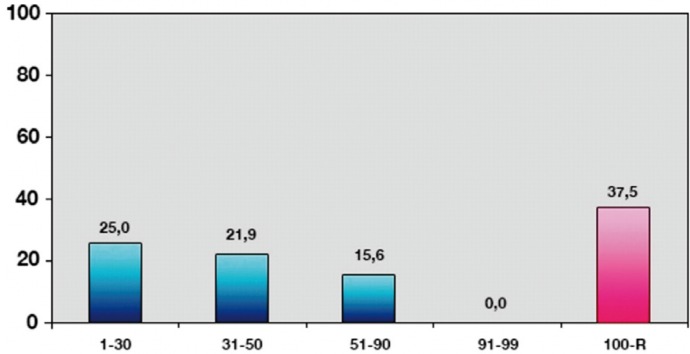
Values obtained by CarieScan system according to intervals proposed by manufacturer.

The results obtained with the CarieScan system are grouped according to the instructions of the manufacturer (Fig. 1).

The CarieScan data were closely correlated to the biopsy findings, with a median of 19.0 among the teeth without caries according to the biopsy findings and a median of 66.5 among the teeth with caries. This difference was significant according to both the Mann-Whitney U-test and the Kruskal-Wallis test (*p* < 0.001). In the absence of biopsy lesions, 50% of the CarieScan readings were between 17-21. In the case of lesions confined to the enamel layer, 50% of the readings were between 22-50, while dentinal lesions yielded readings above 58. The area under the ROC curve was 0.973, showing significant differentiation from the main diagonal. The cutoff point of 21.5 was the optimum value in our study. The sensitivity was found to be 92.9%, with a specificity of 75%, positive predictive value of 96.3%, and a negative predictive value of 60%. In this regard, the method was found to offer high sensitivity and specificity, but with some shortcomings in predicting healthy teeth.

The VistaProof findings were closely correlated to the biopsy results, with a median of 1.0 among the teeth without caries according to the biopsy findings and a median of 2.0 among the teeth with caries ( Table 3). This difference was significant according to both the Mann-Whitney U-test and the Kruskal-Wallis test (*p* < 0.001 and *p* = 0.002, respectively). In the absence of biopsy lesions, 50% of the VistaProof readings were between 0-1.2, approximately. In the case of lesions confined to the enamel layer, 50% of the readings were between 1.6-1.75, while dentinal lesions yielded readings between 1.7-2.25. The area under the ROC curve was 0.969.

**Table 3:**
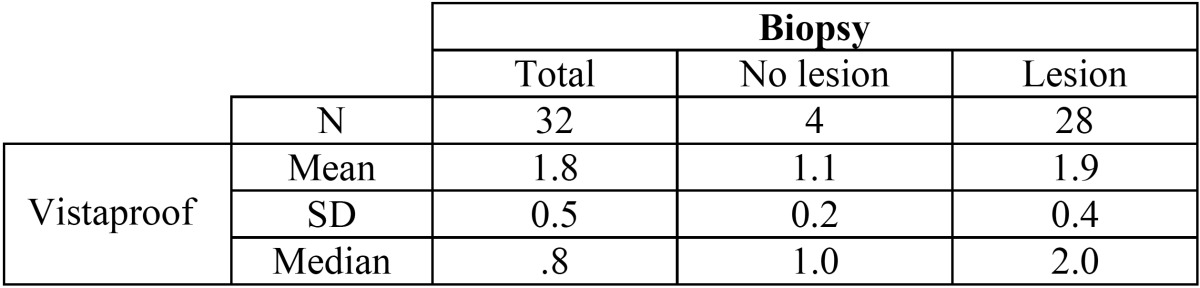
Relationship between the values obtained with the VistaProof method and the actual extent of the lesion.

With a cut off point of 1.1, the sensitivity and negative predictive value were maximum (100%), with a specificity of 75% and a positive predictive value of 96.6%.

## Discussion

The classification used for the histological evaluation was that proposed by Ekstrand (4), and used in other studies found in the literature (5-9).

Regarding visual and tactile inspection, Lussi *et al.* (7) recorded a specificity of 93% with visual inspection, though with a sensitivity of only 12%. In a later “*in vitro*” study, these same authors recorded a sensitivity of 62% with visual inspection when caries already affected the dentin, versus only 31% in the case of lesions confined to the enamel layer (8).

Attrill and Ashley recorded a specificity of over 85% with visual inspection (9,20), while Abalos *et al.* obtained a sensitivity of 93% and a specificity of 88% (5). Goel *et al.* in turn published a comparative study of different diagnostic methods in which relatively low sensitivity (48.1%) was obtained with visual and tactile inspection – though the specificity was 100% (6). Other authors have reported highest sensitivity values with the visual method (18). In our study the visual and tactile diagnoses had the lowest sensitivity performance of all the analyzed techniques (75% and 42.9%, respectively). In this regard, fundamentally the tactile method should be ruled out as a technique for detecting caries, since it showed the poorest performance. The magnitude of the lesion as assessed by visual inspection in turn is scantly consistent with the true extent of the lesion as determined from the biopsy, and so this technique likewise does not offer guarantees in diagnosing caries. The poor sensitivity obtained with the tactile method should cause us to question the usefulness of probing for identifying pit and fissure caries. In this regard, adamantine caries are initially sub-superficial, and the exploratory probe cannot access the fissures. These shortcomings have also been commented by other investigators (1-2,6,11).

A number of authors consider cavity aperture to be the gold standard (8,16,17), while others prefer to base the diagnosis on clinical examination. In our series visual inspection was one of the compared methods, and so could not be taken as a reference.

In our study the DIAGNOdent system offered a sensitivity of 96.4% and a specificity of 100% with a cutoff point of 22.5. Many studies have reported high sensitivity values within a narrow range (79-100%) (12,18,19). These high sensitivity ratings are accompanied by more variable specificity performance, however. The specificity of the laser-induced fluorescence technique applied to dentinal caries in permanent teeth is in the range of 50-100% (12,18,21,22). In the study published by Lussi *et al.* (8), in which dentinal caries was the cutoff point, the sensitivity and specificity of the DIAGNOdent method was 92% and 86%, respectively. However, when enamel caries was taken as the cutoff point, the sensitivity was found to be approximately 96%. Anttonen *et al.* (17) in turn recorded a sensitivity of 92% and a specificity of 82% when using a cutoff point of 30.

The medium in which the samples are stored before evaluation produces a decrease in fluorescence secondary to fluorophore loss in the second week of storage (23). This results in lower DIAGNOdent readings, favoring specificity - an aspect to be taken into account in “in vitro” studies, but not in our “*in vivo*” series. In this regard, “*in vivo*” studies assessing the detection of caries that already affect the dentinal layer have reported results similar to those obtained by “*in vitro*” studies (8,12,18).

The Kappa coefficient has been used to measure the concordance of categorical data. In this respect different studies have obtained values of 0.75-0.98 (16-18), indicating good to excellent agreement, in coincidence with our own findings (Kappa coefficient 0.76).

In the case of the CarieScan method we recorded a sensitivity of 92.9%. In comparison, Ashley *et al.* (9) obtained poorer sensitivity values. The recorded specificity of 75% is very similar to that obtained by other investigators (9,24). The positive predictive value (96.3%) and negative predictive value (60%) recorded in our series reflect high sensitivity and specificity, though with some shortcomings in predicting healthy teeth. Accordingly, the CarieScan system can be regarded as reliable in the case of positive results, though its reliability in the case of negative findings is not so good as when the aforementioned methods are used.

Ismail *et al.* monitored dental caries using a probability analysis of “in vitro” studies, and concluded that this approach may be useful for monitoring changes in the stage of occlusal and other caries (25).

The manufacturer classifies the possible results into ranges: 1-30, 31-50, 51-90, 90-99. In our study, values above 58 were already indicative of dentinal involvement to one degree or other. This differs from the indications of the manufacturer, whereby readings of between 51-90 indicate caries still confined to the enamel layer and possibly extending to the amelodentinal junction.

In our study the VistaProof method offered maximum sensitivity and negative predictive values (100%), with a specificity of 75%. These values are within the ranges described by other investigators such as Diniz *et al.* (14), who reported a specificity of 80% and 74%, respectively, and a sensitivity of 90% and 85%, respectively. Souza *et al.* in turn recorded higher sensitivity and specificity values than with the DIAGNOdent method (26), though other authors have obtained similar results with both techniques (15).

In general, the fluorescence-based systems can be regarded as the most effective techniques, particularly as regards the detection of caried teeth and the reliability of a positive test result.
